# Effects of Phenoxazine Chromophore on Optical, Electrochemical and Electrochromic Behaviors of Carbazole–Thiophene Derivatives

**DOI:** 10.3390/polym16243546

**Published:** 2024-12-19

**Authors:** Bin Hu, Haizeng Song, Xinlei Zhang, Yuan He, Jingshun Ren, Jingbin Huang

**Affiliations:** Henan Key Laboratory of Rare Earth Functional Materials, The Key Laboratory of Rare Earth Functional Materials and Applications, Zhoukou Normal University, Zhoukou 466001, China; songhaizeng0501@foxmail.com (H.S.); xinleizh@126.com (X.Z.); 15737557165@163.com (Y.H.); 15137029765@163.com (J.R.)

**Keywords:** conjugated polymer, electrochromism, phenoxazine chromophore, spectroelectrochemistry

## Abstract

Phenoxazine, as an organic-small-molecule chromophore, has attracted much attention for its potential electrochromic applications recently. To develop appealing materials, phenoxazine chromophores were introduced at the N-position of carbazole–thiophene pigment, yielding two novel monomers (DTCP and DDCP), whose chemical structures were characterized by NMR, HRMS and FTIR. The results of the optical property study indicate that little influence could be observed in the presence of the phenoxazine chromophore. Corresponding polymer films on the surface of an ITO/glass electrode were obtained through electropolymerization. The electrochemical features displayed were various due to the introduction of the phenoxazine group. The spectroelectrochemical results demonstrate that the color of the polymer films could be changed. Compared with the PDDC films, the PDDCP films exhibited three different colors (tangerine, green and purple colors) in different redox states, which could be attributed to the synergistic effect between the carbazole–thiophene conjugate chain and the phenoxazine group. Moreover, fast switching time could be seen due to the presence of the phenoxazine chromophore. This study could provide a reference for obtaining high-performance electrochromic materials.

## 1. Introduction

Electrochromic materials can exhibit reversible color changes under different applied potentials, making them promising for applications in displays, smart windows, information storage and military camouflage [1,2,3,4]. Three types of electrochromic materials have been reported according to previous studies [5,6,7]. Compared with inorganic oxides, organic small molecules and conjugated polymers attract significant attention due to advantages such as easier modification, better color-tuning ability, lower cost and better flexibility [8,9,10]. In organic small molecules, color change is achieved by using solution-based systems or devices based on solutions or gels. However, forming films is difficult to achieve due to their high solubility in common organic electrolytes [11,12,13]. Conjugated polymer film can be more easily prepared through spin coating or electropolymerization [14,15]. Incorporating organic small molecules into conjugated structures is an interesting approach to exploring their effects on optical and electrochemical properties.

Carbazole and its derivatives have been widely used in the optoelectronic field due to their good carrier transport properties and high triplet energy levels [16,17,18]. Carbazole possesses many active sites (3,6-position, 2,7-position and N-position), which makes it a good candidate for investigating the relationship between structure and performance. Wu’s group prepared five polymeric membranes based on the carbazole–thiophene unit. The 3,6-linked conjugated polymer films displayed good electrochemical properties, fine-tuned color ability and fast switching time [19]. Wang’s group synthesized two carbazole–EDOT electrochromic conjugated polymers by linking different positions (2,7 vs. 3,6). The results indicated that the 3,6-linked polymer films possessed lower molecular weight than the 2,7-based polymer films, which resulted in better electrochemical stability of the 2,7-linked polymer films [20]. In our recent study [21], four conjugated microporous polymer films based on carbazole–thiophene were designed and synthesized. The 2,7-linked polymer films also exhibited good electrochromic performance. It is worth exploring the optical and electrochemical features of 2,7-linked carbazole derivatives in detail.

Phenoxazine, as a heterocyclic chromophore, integrates electron-rich nitrogen and oxygen atoms, which leads to a dihedral angle between the two benzene rings in its chemical structure [22,23]. Moreover, the oxygen atom shows stronger electron-pulling ability than the nitrogen atom. Integrating this chromophore is likely to result in lower oxidation potential and enhanced long-term stability [24]. Constantin’s group designed and synthesized three polymers based on triarylamine units, with phenoxazine as side-chain groups. The results indicated that the presence of phenoxazine benefits the stability and efficiency of electrochromic materials [25]. Zhang’s group synthesized two carbazole derivatives by introducing the phenothiazine/phenoxazine group into the main chain linked by alkyl. Although the corresponding polymer films exhibited good electrochemical and electrochromic features, the effect of the phenothiazine/phenoxazine group was not so obvious [26]. In our previous work [27], three 2,5-di(2-thienyl)-1H-pyrrole-based polymer films were prepared with different phenoxazine units as side chains, linked though a conjugated structure. The electrochemical and color-changing abilities were significantly influenced by the solubility of the obtained polymer film. Copolymers were used to enhance their performance, though this weakened the effect of the side-chain groups. It is interesting to explore the effect of phenoxazine as a side-chain group in a conjugated structure.

**Scheme 1:**
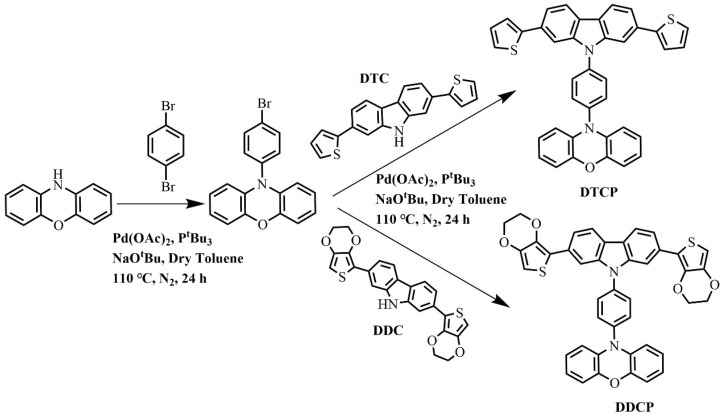
Synthesis routes of DTCP and DDCP.

According to the above analysis, two novel monomers based on carbazole–thiophene and the phenoxazine chromophore were designed and synthesized (Scheme 1). The corresponding polymer films were prepared via electropolymerization. The effect of phenoxazine units on the optical, electrochemical and electrochromic properties were investigated and discussed in detail.

## 2. Materials and Methods

### 2.1. Materials

Phenoxazine (98%), 1,4-dibromobenzene (99%), palladium(II) acetate (Pd(OAc)_2_; 99%), sodium tert-butoxide (NaOt-Bu; 98%) and tri-tert-butylphosphine (P(t-Bu)_3_; 10 *w*/*v*% in Toluene) were purchased from Energy Chemical (Shanghai, China). 2,7-Di(thiophen-2-yl)-9H-carbazole (DTC) and 2,7-bis(2,3-dihydrothieno[3,4-b][1,4]dioxin-5-yl)-9H-carbazole (DDC) were obtained from our reported studies [21], and toluene and dichloromethane (DCM) were dried with CaH_2_ for about 72 h and were then collected and saved with molecular sieves (4 Å). Acetonitrile (ACN; 99.99% trace metals basis) was purchased from Aladdin (Shanghai, China) and used without further purification. Tetrabutylammonium perchlorate (TBAP; 98%) was purchased from Zhengzhou Alfa Chemical Co., Ltd. (Zhengzhou, China). The other regents were commercial products used as received.

### 2.2. Measurements

A Bruker Avance NEO (Bruker, Saarbruecken, Germany) was used to investigate the NMR spectra (^1^H and ^13^C) of the obtained compounds. Q Exactive Orbitrap (Thermo Fisher Scientific, Waltham, MA, USA) was used to investigate the high-resolution mass spectrometry (HRMS)-derived spectra of the prepared monomers. Nicolet iS20 (Thermo Fisher Scientific, Waltham, MA, USA) was used to investigate the infrared spectrometry properties of the prepared monomers and polymer films. The UV–vis spectra of the obtained monomers and polymer films were recorded by a Lambda 25 UV–vis spectrophotometer (Perkin Elmer, Waltham, MA, USA). The electrochemical properties of the monomers and polymer films were investigated with a Chi660 electrochemical analyzer (Shanghai Chenhua Ins, Shanghai, China). Quanta 200 (FEI, Hillsboro, OR, USA) was employed to measure the morphology of the prepared polymer films.

### 2.3. Synthesis

#### 2.3.1. Synthesis of 10-(4-Bromophenyl)-10H-Phenoxazine

Phenoxazine (0.37 g, 2 mmol) and 1, 4-dibromobenzene (0.47 g, 2 mmol) were placed in a dry flask with three necks; then, Pd(OAc)_2_ (0.03 g), NaOt-Bu (0.19 g) and 15 mL of dry toluene were also added, and the mixture was placed in a magnetic stirrer and stirred for five minutes; finally, the mixture was degassed by using a Schlenk system, and 4 mL of P(t-Bu)_3_ was added with a syringe. The mixture was stirred at 110 °C for 24 h in a N_2_ atmosphere. After being cooled to room temperature, the mixture was processed by a rotary evaporator to remove the toluene and was then subjected to extraction with DCM and supersaturated salt water. The organic phase was gathered and dried with anhydrous Mg_2_SO_4_. After filtration, the obtained solvent was removed under vacuum pressure, and the residue was processed by column chromatography on silica gel with petroleum: DCM (10:1 by volume) to give a bright powder (0.46 g, 67%). ^1^H NMR (400 MHz, CDCl_3_) *δ*: 7.72 (d, 2H), 7.23 (d, 2H), 6.64 (m, 6H), 5.91 (d, 2H). ^13^C NMR (100 MHz, CDCl_3_) *δ*: 143.95, 138.14, 134.46, 134.02, 132.77, 123.31, 122.39, 121.65, 115.61, 113.21. HRMS (APCI) (*m*/*z*): calcd for C_18_H_12_BrNOS: 338.02; found: 338.0484.

#### 2.3.2. Synthesis of 10-(4-(2,7-Di(Thiophen-2-Yl)-9H-Carbazol-9-Yl)Phenyl)-10H-Phenoxazine (DTCP)

10-(4-Bromophenyl)-10H-phenoxazine (0.38 g, 1 mmol) and DTC (0.31 g, 1 mmol) were placed in a dry flask with three necks; then, Pd(OAc)_2_ (0.02 g), NaOt-Bu (0.1 g) and 10 mL of dry toluene were also added, and the mixture was placed in magnetic stirrer and stirred for five minutes; finally, the mixture was degassed by using a Schlenk system, and 3 mL of P(t-Bu)_3_ was added with a syringe. The mixture was stirred at 110 °C for 24 h in a N_2_ atmosphere. After being cooled to room temperature, the mixture was processed by a rotary evaporator to remove the toluene and was then subjected to extraction with DCM and supersaturated salt water. The organic phase was gathered and dried with anhydrous Mg_2_SO_4_. After filtration, the obtained solvent was removed under vacuum pressure, and the residue was processed by column chromatography on silica gel with petroleum: DCM (5:1 by volume) to give a light-yellow powder (0.33 g, 56%). ^1^H NMR (400 MHz, CDCl_3_) *δ*: 8.08 (d, 2H), 7.83 (d, 2H), 7.63 (dd, 4H), 7.58 (d, 2H), 7.35 (d, 2H), 7.29 (d, 2H), 7.09 (t, 2H), 6.73 (m, 6H), 6.13 (t, 2H). ^13^C NMR (100 MHz, CDCl_3_) *δ*: 145.16, 144.12, 141.64, 138.28, 137.37, 134.21, 132.80, 129.47, 128.16, 124.97, 123.45, 122.92, 121.78, 120.85, 119.52, 115.73, 113.42, 107.03. HRMS (APCI) (*m*/*z*): calcd for C_38_H_24_N_2_OS_2_: 588.13; found: 589.1384.

#### 2.3.3. Synthesis of 10-(4-(2,7-Bis(2,3-Dihydrothieno[3,4-b][1,4]Dioxin-5-yl)-9H-Carbazol-9-yl)Phenyl)-10H-Phenoxazine (DDCP)

The synthesis procedure of DDCP was similar to that of DTCP and a green powder was obtained (0.37 g, 53%). ^1^H NMR (400 MHz, CDCl_3_) *δ*: 8.08 (d, 2H), 7.83 (d, 2H), 7.63 (dd, 4H), 8.07 (d, 2H), 7.88 (d, 2H), 7.63 (dd, 2H), 6.70 (m, 6H), 6.31 (s, 2H), 6.11 (d, 2H), 4.26 (dd, 8H). ^13^C NMR (100 MHz, CDCl_3_) *δ*: 144.12, 142.35, 141.44, 138.05, 137.73, 134.34, 132.38, 131.22, 129.50, 123.32, 122.26, 121.70, 120.48, 119.21, 118.28, 115.72, 113.25, 107.21, 97.68, 64.85, 64.49. HRMS (APCI) (*m*/*z*): calcd for C_42_H_28_N_2_O_5_S_2_: 704.14; found: 705.1486.

### 2.4. Electrochemical Measurements

Electrochemical tests were performed in a three-electrode cell with an ITO/glass (2 cm × 1 cm) as the working electrode, and a platinum sheet and a double-junction Ag/AgCl electrode as the counter electrode and the reference electrode, respectively. Before the tests, the solution in the three-electrode cell was processed by introducing N_2_ gas from the bottom with a three-way glass tube for half an hour, and the following tests were performed at room temperature (about 25 °C).

## 3. Results and Discussion

### 3.1. Optical Properties

**Figure 1:**
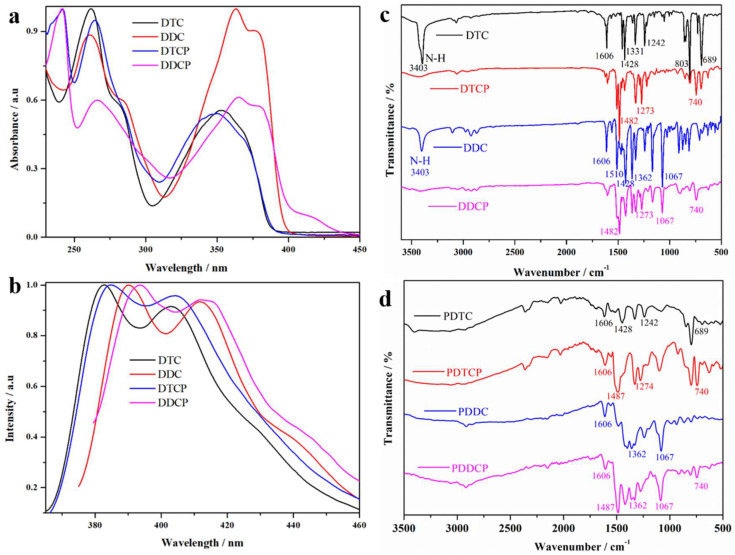
(**a**) The UV−vis absorption and (**b**) emission spectra of DTC, DDC, DTCP and DDCP in DCM solution; the FTIR spectra of (**c**) monomers and (**d**) their corresponding polymer films.

To investigate the influence of phenoxazine units on the optical properties of carbazole–thiophene derivatives, the absorption and fluorescence emission nature of the intermediates and monomers were explored, and the results are shown in Figure 1a,b. For DTC and DDC, the absorption peak around 260 nm can be attributed to the core of the carbazole unit, the peaks located at 352 and 360 nm can be ascribed to the π-π* transition of the carbazole–thiophene fragment [28], and the red shift phenomenon can be explained by the fact that EDOT units possess better donor ability than the thiophene units. After the introduction of the phenoxazine units, a new absorption peak at 242 nm appeared, which can be the π-π* transition of 10-(4-phenyl)-10H-phenoxazine [25]. Moreover, the absorption features at 260 and 350 nm of DTCP and DDCP showed little change compared with those of DTC and DDC. A similar phenomenon was found for the emission features (Figure 1b); the effect of the presence of 10-(4-phenyl)-10H-phenoxazine at the side chain was less unobvious than that of the introduction of the EDOT units at the main chain. Both DTCP and DDCP exhibited blue color emission.

### 3.2. FT-IR Characterization

FT-IR was used to characterize the chemical structure of monomers and their corresponding polymer films, which were processed in a vacuum oven at 60 °C in a vacuum environment for 12 h before the tests, and the results are shown in Figure 1c,d. As seen in Figure 1c, the absorption peak at 3403 cm^−1^ can be attributed to the stretching vibration of the N-H bond of the carbazole units for DTC and DDC, and the stretching vibration of the C=C bond can be seen at 1606 cm^−1^; the absorption peak at 1428 cm^−1^ can be ascribed to the deformation vibration of the C-H bond of the carbazole units, whose out-of-plane bending vibration of the C-H bond was found at 803 cm^−1^. The absorption waves at 1331 and 1242 cm^−1^ are attributed to the in-plane bending vibration of the C-H of the thiophene groups, and the out-of-plane bending vibration of the C-H bond was observed at 689 cm^−1^ [29]. For DDC, some new absorption peaks appeared: that at 1510 cm^−1^ is ascribed to the stretching vibration of the C-C bond of the EDOT units, and the absorption waves at 1362 and 1067 cm^−1^ can be attributed to the vibration of the C-O-C bond [30]. After the introduction of phenoxazine units, the absorption peaks at 3403 cm^−1^ disappeared for both DTCP and DDCP, indicating that the N-H bonds had been replaced. Further, for DTCP, the new absorption peaks at 1482 and 1273 cm^−1^ can be attributed to the vibration of the C=C and C-O bonds of the phenoxazine group, and the obvious peak at 740 cm^−1^ belongs to the bending vibration of the C-H bond of the phenoxazine group [31]. DDCP displayed similar absorption features to DDC, confirming that the target monomers were obtained.

Figure 1d shows the FT-IR spectra of the resulting polymer films via electropolymerization. All the polymer films exhibited non-obvious sharp peaks compared with the monomers but retained the characteristic features; however, some differences were observed: the bending vibration of the C-H of the thiophene groups at 689 cm^−1^ became weak, indicating that polymerization occurred at the α position of the thiophene ring [32]. Compared with PDTC, PDTCP displayed obvious absorption peaks at 1487, 1274 and 740 cm^−1^, which belong to the characteristic peaks of phenoxazine units. For PDDC and PDDCP, similar features were also observed, and the vibration of the C-O-C bond of the EDOT group at 1362 cm^−1^ was still obvious. All the results indicate that the designed polymer films were achieved.

### 3.3. Electrochemical Properties

**Figure 2:**
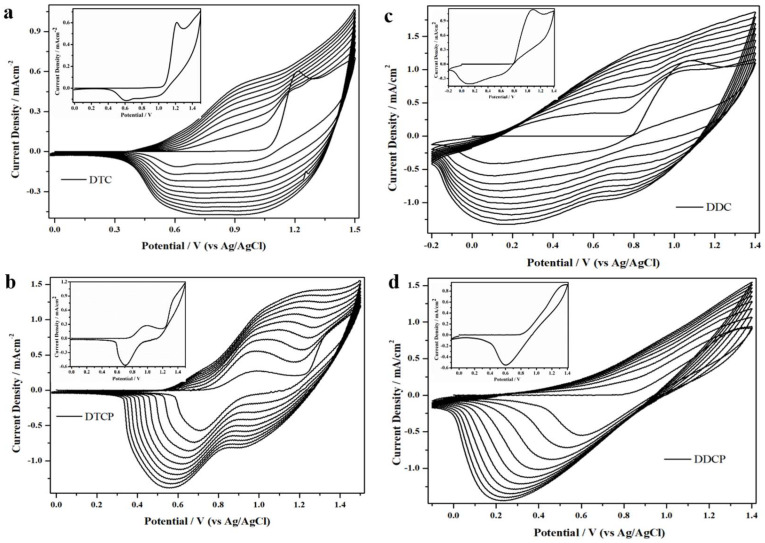
Continuous CV electropolymerization curves of (**a**) 1 mM DTC, (**b**) 1 mM DTCP, (**c**) 1 mM DDC and (**d**) 1 mM DDCP in the 0.1 M TBAP/DCM solution at a scan rate of 100 mV/s (inset images: the first cycle of CV curves).

To investigate the electrochemical features and obtain the desired polymer film, continuous cyclic voltammetry (CV) was carried out in the three-electrode cell with a DCM solution of 1 mM monomer and 0.1 M TBAP as the supporting electrolyte at a scan rate of 100 mV/s, and the results are shown in Figure 2. All the intensities of the current increased with the increase in scan cycles, indicating that polymerization occurred on the surface of the ITO/glass electrode [33]; further, the polymer films could be observed with the naked eye, and the reversible colors of the polymer films changed with the shift in the applied potentials. All the CV curves were reversible except for the first cycle, which is ascribed to the process of the generation of free radicals [34]. As seen from the inset images in Figure 2, for DTC, the oxidation and reduction peaks can be found at 1.22 and 0.61 V, and the oxidation position can be ascribed to the thiophene ring of the carbazole–thiophene conjugated structure; the onset oxidation potential was calculated to be 1.06 V. DDC shows the oxidation and reduction peaks at 1.08 and 0.12 V, and the lower oxidation potential can be ascribed to the better donor ability of EDOT units; the onset oxidation potential was found to be 0.79 V. After the introduction of phenoxazine units, DTCP exhibited two oxidation waves at 0.98 and 1.32 V, with an obvious reduction peak located at 0.71 V. DDCP showed the oxidation and reduction peaks at around 1.31 and 0.61 V, and the onset oxidation potentials of DTCP and DDCP were calculated to be 0.78 and 0.81 V. The differences from those of DTC and DDC indicate that the introduction of phenoxazine units had an influence on the electrochemical properties.

**Figure 3:**
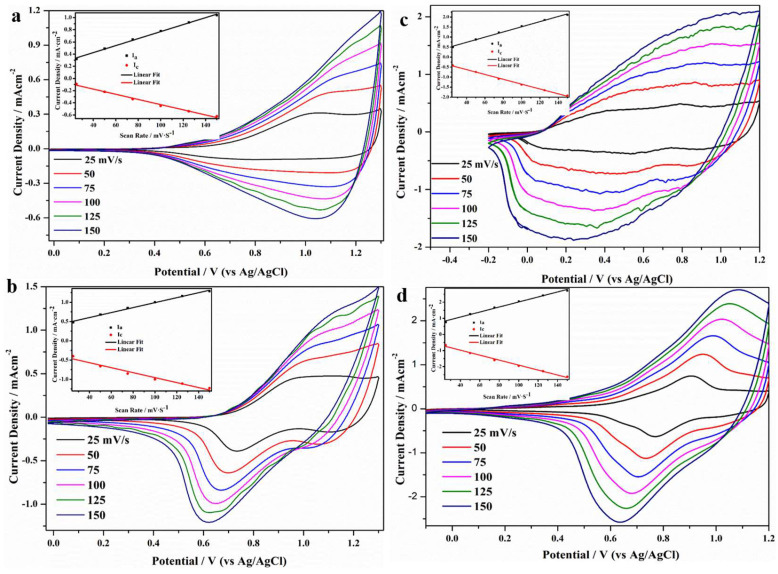
CV curves of (**a**) PDTC, (**b**) PDTCP, (**c**) PDDC and (**d**) PDDCP films in monomer-free solution of 0.1 M TBAP/ACN at different scan rates from 25 to 150 mV/s with 25 mV increments (inset images: the linear relationship between anodic/cathodic current intensity and scan rate).

The electrochemical properties of polymer films were also explored, and the results can be seen in Figure 3. Different electrochemical features were observed compared with those of the monomers. The PDTC films showed oxidation and reduction waves at 1.04 and 1.05 V at the scan rate of 25 mV/s. Thanks to the presence of the EDOT units, PDDC displayed a broader electrochemical range, with the oxidation and reduction peaks at around 0.79 and 0.54 V. The introduction of the phenoxazine group made the PDTCP and PDDCP films show similar electrochemical properties, and the oxidation (around 0.95 V) and reduction (0.75 V) waves could be observed for both the PDTCP and PDDCP films at the scan rate of 25 mV/s, indicating that the phenoxazine group had a great effect on the electrochemical properties of polymer films. Moreover, the intensity of the current gradually increased with the increase in the scan rate, and the linear relationship between peak current and scan rate (inset of Figure 3) could be estimated, indicating that all the redox events were controlled by electron transfer kinetics on the surface of the ITO/glass electrode rather than the diffusion process [35].

### 3.4. Morphology of Polymer Films

**Figure 4:**
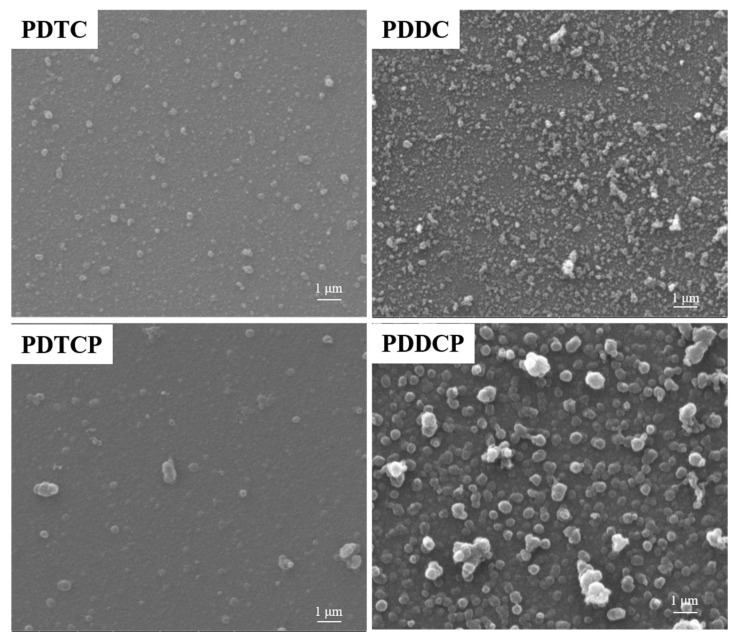
The micromorphology images of the PDTC, PDTCP, PDDC and PDDCP films.

The relationship between microstructure and performance is worth exploring, so a scanning electron microscope (SEM) was used to investigate the morphology. Figure 4 shows the microstructure of the polymer films. As seen from Figure 4, the PDTC and PDDC films showed unevenly sized particles on the surface, and the size of the particles for PDTC was smaller, which can be the effect of different thiophene derivatives. After the introduction of the phenoxazine group, a more even surface can be observed for PDTCP and PDDCP films, and the size was bigger than that of the corresponding carbazole–thiophene intermediate; however, some aggregation still exists. The differences can be ascribed to the influence of the presence of the phenoxazine group, which may have some effect on electrochromic performance [36].

### 3.5. Spectroelectrochemistry

**Figure 5:**
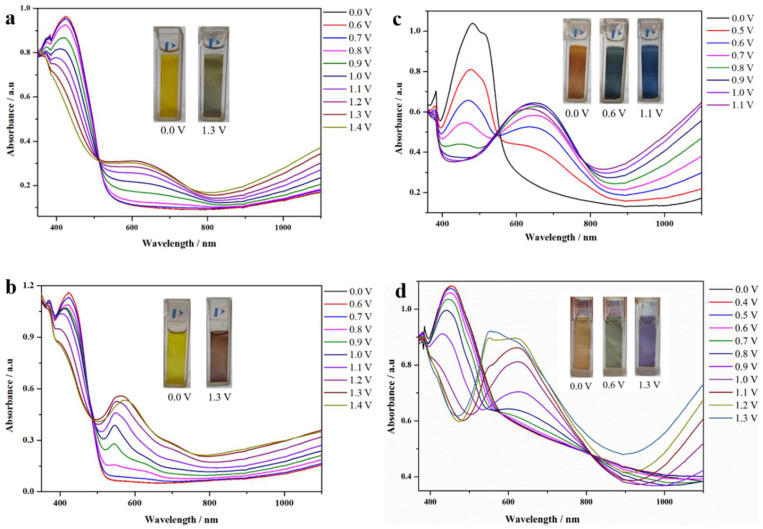
Spectroelectrochemical spectra of (**a**) PDTC, (**b**) PDTCP, (**c**) PDDC and (**d**) PDDCP films under different applied potentials in 0.1 M TBAP/ACN solution (inset picture: images of polymer films under different applied potentials).

**Figure 6:**
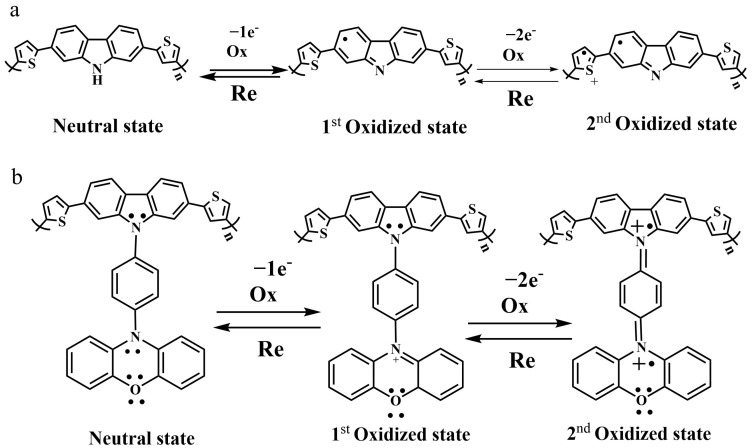
The proposed electrochromic mechanisms of (**a**) PDTC and (**b**) PDTCP films in different oxidized states.

The change in color is due to the doping effect of the conjugated structure under different applied potentials, and such effect can be monitored via spectroelectrochemistry by using a UV–vis spectrophotometer and an electrochemical station simultaneously; the results can be seen in Figure 5. The PDTC films showed a maximum absorption peak at 423 nm in the neutral state (Figure 5a), which can be attributed to the π-π* transition of the conjugated chain based on carbazole and thiophene [37]. The onset absorption wavelength was 537 nm, and this absorption feature made the PDTC films display an orange color; according to the reported calculated methods [38], the band gap of the PDTC films in the neutral state was found to be 2.31 eV. The presence of the EDOT group made the maximum absorption peak of the PDDC films redshift to 483 nm for the neutral state due to better donor ability. The onset absorption wavelength was 624 nm (Figure 5c), and the corresponding bang gap was calculated to be 1.99 eV; brick red color can be observed at 0 V. After the introduction of the phenoxazine group, the PDTCP and PDDCP films exhibited maximum absorption waves at 422 and 455 nm, respectively; the corresponding onset absorption wavelengths were found to be 519 and 590 nm (Figure 5b,d), and their band gap (2.39 and 2.10 eV) were obtained. The color displayed by the PDTCP and PDDCP films in the neutral state were yellow and tangerine. By contrast, the presence of the phenoxazine group had a larger influence on the carbazole–EDOT segments; the reason may be that carbazole–EDOT segments possess more electron-rich regions, which are prone to be affected. With the increase in applied potential, the absorption curves of all the polymer films changed due to the doping effect. For PDTC and PDDC, the absorption waves around 630 and 1100 nm became gradually stronger thanks to the generation of polarons and bipolarons [38]; during the oxidation process, isosbestic points could observed, and the PDTC and PDCC films showed these points at 513 and 560 nm, respectively. Further, the PDTC films showed grayish green color in the full oxidation state (1.30 V), and blue-green (0.60 V) and blue (1.10 V) colors could be seen for the PDDC films. Due to the generation of polarons and bipolarons, the PDTCP (or PDDCP) films exhibited new absorption at around 544 (or 546 nm) and 1100 nm, and the difference from PDTC or PDDC can be attributed to the effects of the introduction of phenoxazine units [25]. The isosbestic points at 486 and 557 nm for PDTCP and PDDCP were found, and the difference from PDTC and PDDC can be ascribed to the introduction of phenoxazine units. Compared with the absorption feature for the neutral state, the influence on the absorption curve of carbazole–thiophene was larger for the oxidation state, and the reason may be that electron-rich carbazole–EDOT segments produce stronger absorption than phenoxazine units in the oxidation state [39]. Such absorption features made the polymer films show different colors under different applied potential (light red (1.3 V) for PDTCP; green (0.6 V) and purple (1.3 V) for PDDCP). During the oxidation process, the doping effect led to a change in the chemical structure of the polymer films, and the proposed mechanisms of PDTC and PDTCP films in different oxidized states are shown in Figure 6. The presence of the phenoxazine group linked by the conjugated structure can change the chemical structure of the oxidation state, which can be considered an effective way to achieve high-performance electrochromic materials [22,25].

### 3.6. Electrochromic Switching

**Figure 7:**
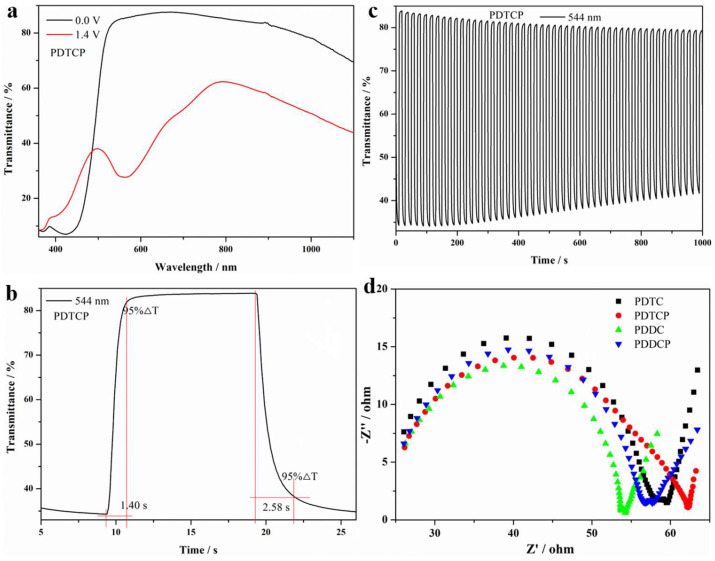
(**a**) The relationship between transmittance and different wavelengths of the PDTCP films at 0.0 and 1.3 V. (**b**) The transmittance–time profiles of the PDTCP films monitored at different absorption maxima under applied potential between 0.0 V and 1.3 V with switching intervals of 10 s. (**c**) The calculated switching time of PDTCP at 544 nm. (**d**) The EIS spectra between Zre and ω^−0.5^ at low frequencies for the PDTC, PDTCP, PDDC and PDDCP films in a monomer-free solution of 0.1 M TBAP/ACN.

Optical contrast, switching time and coloration efficiency (CE) are three important performance parameters for the potential applications of electrochromic materials, which can also be tested by using a UV–vis spectrophotometer and an electrochemical station simultaneously [40]; the results can be found in Figure 7. The optical contrast at the specific wavelength is the transmittance change under two different applied potentials, which can be estimated by the spectral graph of transmittance under different wavelengths (Figure 7a and Figures S3a–S5a), the maximum transmittance of the PDTCP films under different applied potentials was found at 544 nm, and the optical contrast (ΔT) was calculated to be 50%; after 1000 s, the optical contrast decreased to 38% (Figure 7c). The maximum transmittance of the PDTC films was at 610 nm (Figure S5b), ΔT was found to be 43%, and the stability of the polymer in solution was 75% after 1000 s, indicating that the introduction of the phenoxazine group had little influence on optical contrast and stability. A similar phenomenon was also seen for the PDDCP and PDDC films: the values of ΔT were found to be 21% and 27% (Figure S3b and Figure S4b), respectively; the stability values were 81% and 85% after 1000 s, and the improved stability can be attributed to the presence of EDOT units.

The switching time was estimated to be the shift time according to the 95% full-transmittance change [41]. As seen from Figure 7b and Figures S3–S5, both the PDTCP and PDDCP films showed fast switching time (1.40 and 2.58 s for PDTCP (Figure 7b); 1.78 and 2.02 s for PDDCP) (Figure S3c), and the difference between the colored and bleached time was small. Compared with the PDTCP and PDDCP films, PDTC and PDDC exhibited bigger differences (0.92 and 3.39 s for PDTC; 4.37 and 1.08 s for PDDC) (Figures S4c and S5c), indicating that the PDTCP and PDDCP films possessed better quality, which is consistent with the results of the microstructure.

CE can be calculated by the formula CE = ΔOD/Qd (where ΔOD is the change in optical density at the specific wavelength, which can be obtained by the formula ΔOD = lg(T_bleached_/T_colored_), and Qd is the injected/ejected charge as a function of the electrode area) [21]. PDTC and PDTCP showed CEs of 95.40 cm^2^C^−1^ (610 nm) and 126.24 cm^2^C^−1^ (544 nm), and values of 86.34 cm^2^C^−1^ (650 nm) and 96.72 cm^2^C^−1^ (546 nm) were obtained for the PDDC and PDDCP films; these changes can be attributed to the presence of phenoxazine units. These optical and electrochromic properties are summarized in Table 1. Compared with the other carbazole derivatives, the PDTCP and PDDCP films showed reasonable electrochromic properties.

To study the electrochemical process of polymer films in depth, electrochemical impedance spectroscopy (EIS) was used to investigate the ion diffusion phenomenon in the polymer films in solution within the frequency range from 0.1 Hz to 100 KHz, and the results can be seen in Figure 7d. All the polymer film possessed almost the same R_Ω_ due to the same test conditions. Charge transfer resistance (Rct) indicates the rate of transferred charge from electrode to electrolyte; the PDDC films exhibited the lowest Rct (54 ohm), and 59 ohm was found for the PDTC films. The presence of the phenoxazine group increased the charge transfer resistance, indicating that the introduction of the group at the side chain is not good for the charge transferred ability of polymer [42], but such influence is not obvious.

## 4. Conclusions

Two new carbazole–thiophene derivatives containing the phenoxazine group (DTCP and DDCP) were successfully synthesized and characterized. The absorption and fluorescence emission results show that little influence could be seen before and after the introduction of the phenoxazine group. The electrochemical property study indicated that obvious effects could be observed due to the presence of the phenoxazine group. The introduction of the phenoxazine group led to changes in the microstructure of the polymer films. In addition, the spectroelectrochemical results indicate that the PDTC films displayed orange color in the neutral state and changed to grayish green color in the oxidation state. The PDTCP films changed color from yellow to light red in different states. The color of the PDDC films changed from brick red to brown and blue upon potential scanning from 0.0 to 1.3 V. And the PDDCP films exhibited tangerine color in the neutral state and changed to green and purple color in the oxidation state. Though optical contrast and stability showed little change before and after the introduction of the phenoxazine group, faster switching time could be observed thanks to the better quality of the polymer films. The electrochemical impedance spectroscopy study showed that the introduction of the group at the side chain was not good for the charge transferred ability of the polymer. We hope that these findings can provide a reference for achieving high-performance electrochromic materials.

## Figures and Tables

**Table 1:** The optical and electrochromic parameters of carbazole-based polymers.

Polymers	λonset (nm)	Eg * (eV)	ΔT%	t_b_ (s)	t_c_ (s)	CE (cm^2^/C)
PDTC	537	2.31	43% (610 nm)	0.92 (610 nm)	3.39 (610 nm)	95.40 (610 nm)
PDDC	624	1.99	27% (650 nm)	4.37 (650 nm)	1.08 (650 nm)	86.34 (650 nm)
PDTCP	519	2.39	50% (544 nm)	1.40 (544 nm)	2.58 (544 nm)	126.24 (544 nm)
PDDCP	590	2.10	21% (546 nm)	1.78 (546 nm)	2.02 (546 nm)	96.72 (546 nm)
PCz-OH [18]	395	3.14	26% (875 nm)	2.1 (875 nm)		
P(2,7-Cbz-E)-LW [20]	523	2.37	19% (605 nm)	4.6 (605 nm)	0.4 (605 nm)	139 (605 nm)
PM2 [21]	505	2.46	22.91% (560 nm)	0.77 (560 nm)	1.29 (560 nm)	82 (560 nm)

* Calculated by using Eg = 1240/λonset; λonset: the onset value of the absorption peak.

## Data Availability

Data are contained within the article and Supplementary Materials.

## Supplementary Materials

The following supporting information can be downloaded at: https://www.mdpi.com/article/10.3390/polym16243546/s1, Figure S1: The ^1^H and ^13^C NMR spectra of intermediate and monomers (DTCP and DDCP) dissolved in CDCl_3_, Figure S2: The high-resolution mass spectrometry spectra of 10-(4-bromophenyl)-10H-phenoxazine, DTCP and DDCP, Figure S3. The relationship between transmittance and different wavelengths for the PDDCP films at 0.0 and 1.3 V; (b) the transmittance–time profiles of the PDDCP films monitored at different absorption maxima under applied potential between 0.0 V and 1.3 V with a switching time of 10 s; (c) the calculated switching time of PDDCP at 546 nm, Figure S4: The relationship between transmittance and different wavelengths for the PDDC films at 0.0 and 1.3 V; (b) the transmittance–time profiles of the PDDC films monitored at different absorption maxima under applied potential between 0.0 V and 1.3 V with a switching time of 10 s; (c) the calculated switching time of PDDC at 650 nm, Figure S5: The relationship between transmittance and different wavelengths for the PDTC films at 0.0 and 1.3 V; (b) the transmittance–time profiles of the PDTC films monitored at different absorption maxima under applied potential between 0.0 V and 1.3 V with a switching time of 10 s; (c) the calculated switching time of PDTC at 610 nm.
