# Supplementary material for: Effects of Phenoxazine Chromophore on Optical, Electrochemical and Electrochromic Behaviors of Carbazole–Thiophene Derivatives
Source: Polymers (Basel). 2024 Dec 19;16(24):3546. doi: 10.3390/polym16243546 (PMC11678722; doi:10.3390/polym16243546)
Supplement: Supplementary file 1 [file polymers-16-03546-s001.zip › polymers-3338882-supplementary.pdf]

Supporting Materials

Effects of phenoxazine chromophore on the optical, electrochemical and electrochromic behaviors of carbazole-thiophene derivatives

Bin Hu*, Haizeng Song, Xinlei Zhang, Yuan He, Jingshun Ren, Jingbin Huang*

Henan Key Laboratory of Rare Earth Functional Materials; The Key Laboratory of Rare Earth Functional Materials and Applications, Zhoukou Normal University, Zhoukou, 466001, P.R. China. Fax: +86-394-8178518; Tel: +86-394-8178518

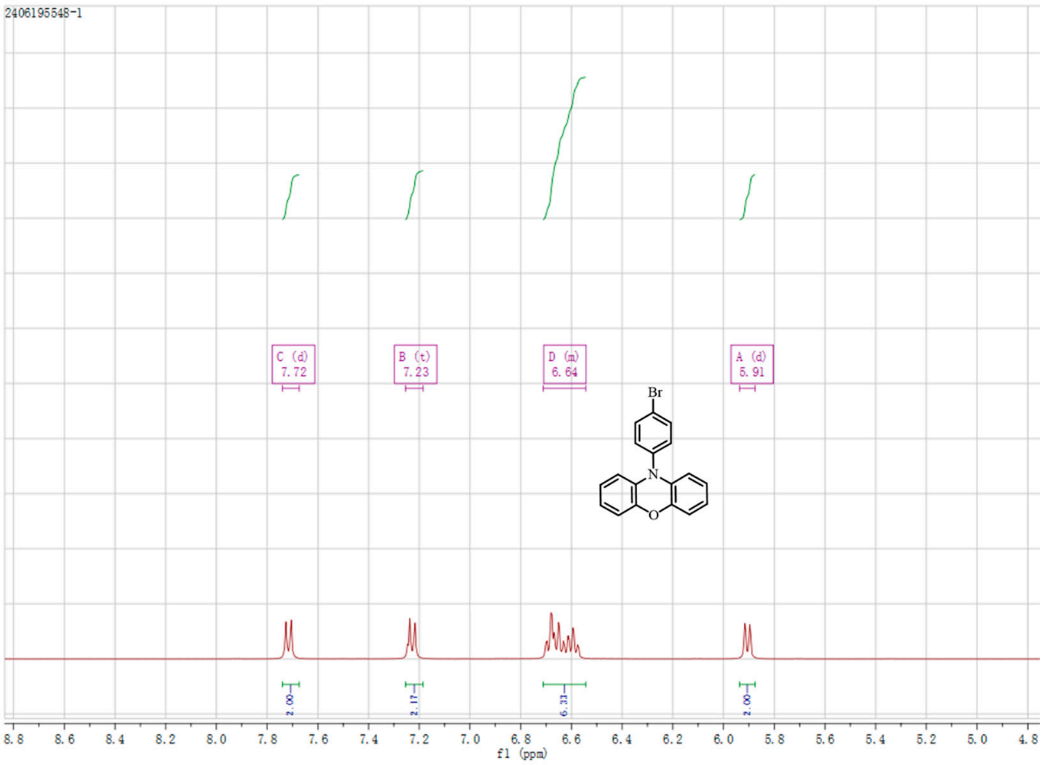

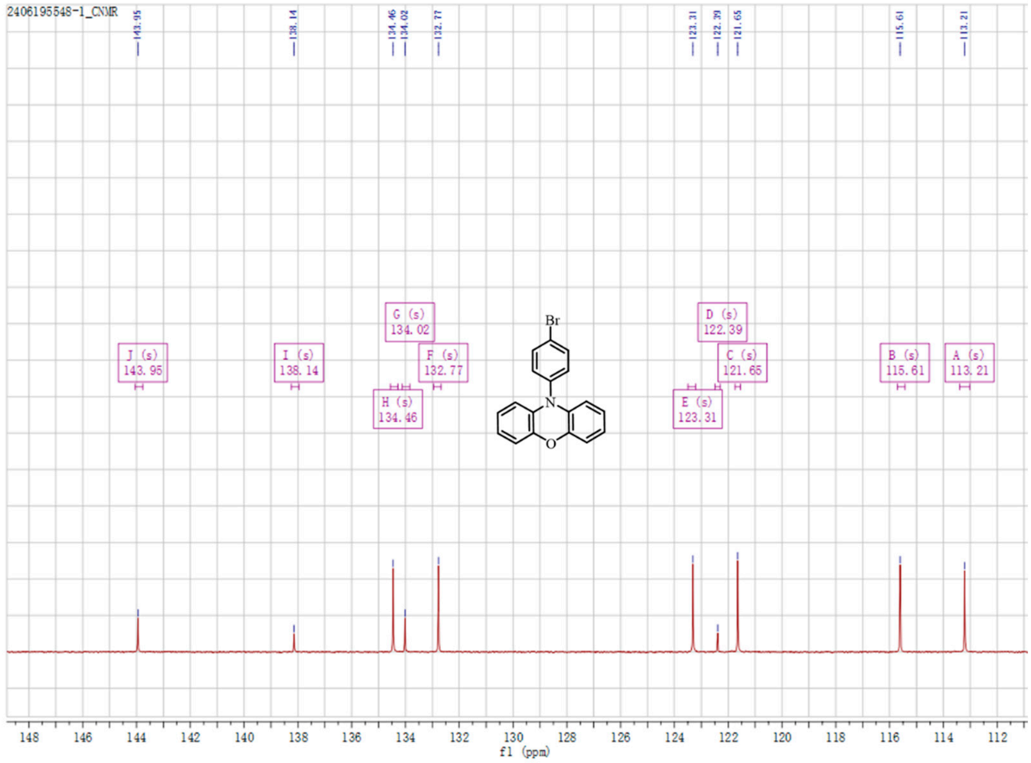
2406195548-1_CNMR
143.95
138.14
134.46
134.02
132.77
123.31
122.39
121.65
115.61
113.21
J (s) 143.95
I (s) 138.14
G (s) 134.02
H (s) 134.46
F (s) 132.77
D (s) 122.39
E (s) 123.31
C (s) 121.65
B (s) 115.61
A (s) 113.21
Br
N
O
148 146 144 142 140 138 136 134 132 130 128 126 124 122 120 118 116 114 112
f1 (ppm)

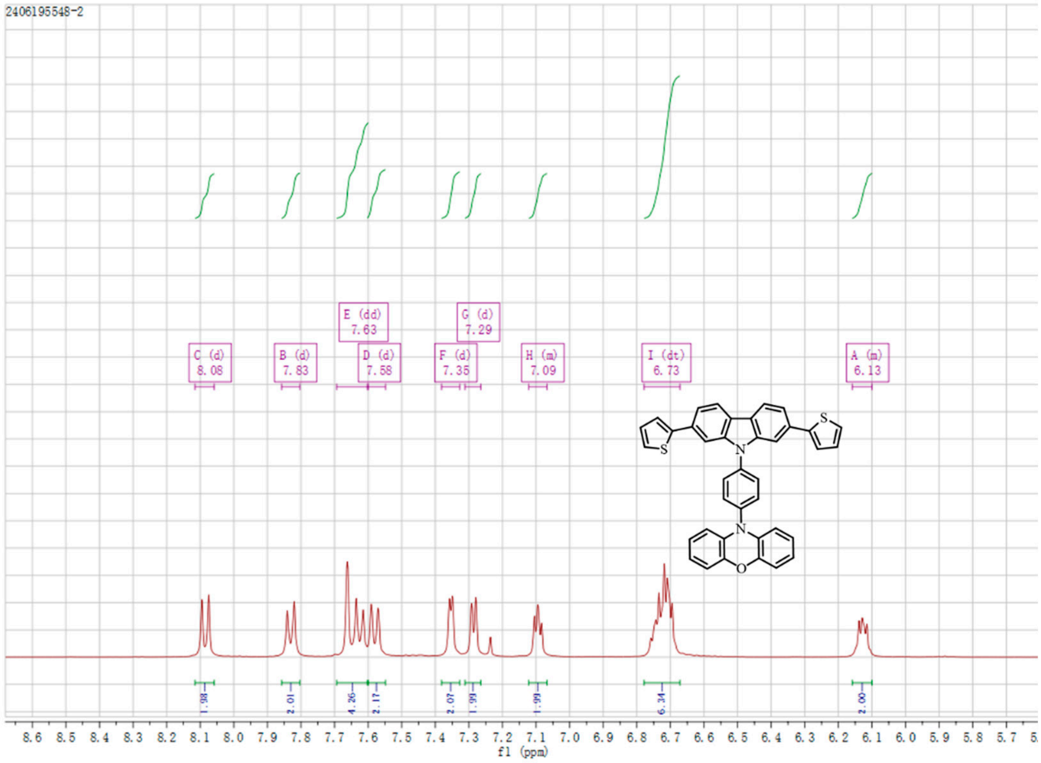
2406195548-2
E (dd) 7.63
G (d) 7.29
C (d) 8.08
B (d) 7.83
D (d) 7.58
F (d) 7.35
H (m) 7.09
I (dt) 6.73
A (m) 6.13
S
N
O
1.98
2.01
4.26
2.17
2.07
1.99
1.99
6.34
2.00
8.6 8.5 8.4 8.3 8.2 8.1 8.0 7.9 7.8 7.7 7.6 7.5 7.4 7.3 7.2 7.1 7.0 6.9 6.8 6.7 6.6 6.5 6.4 6.3 6.2 6.1 6.0 5.9 5.8 5.7
f1 (ppm)

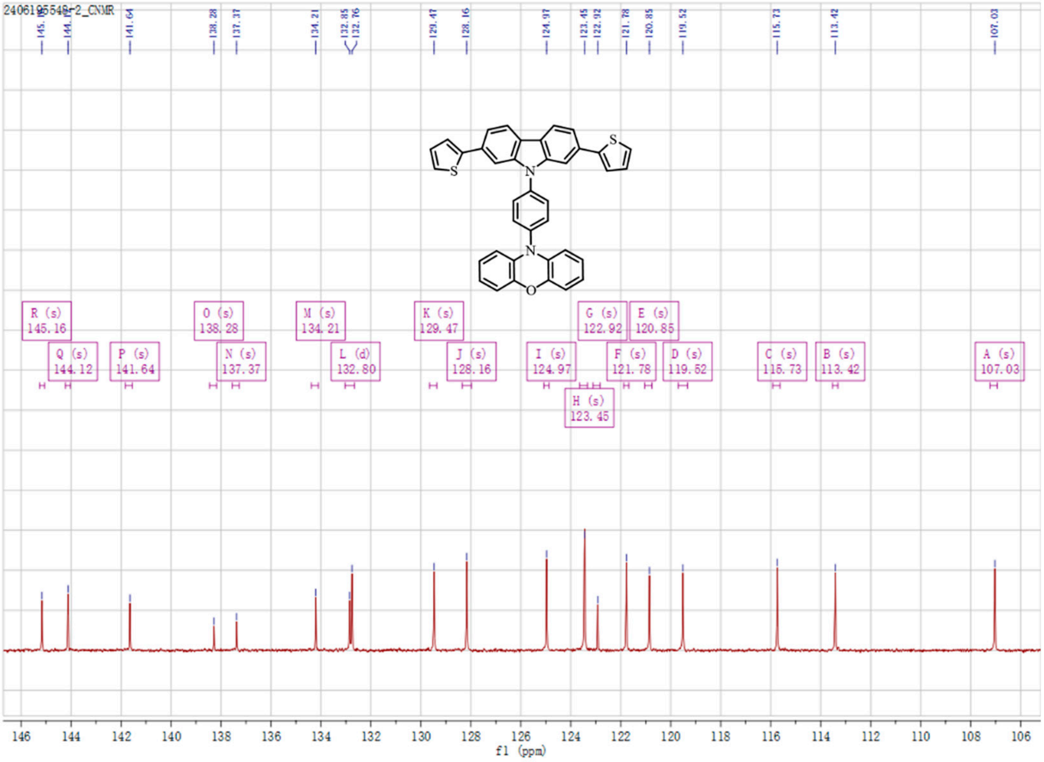

2406195548-2_CNMR
R (s) 145.16
Q (s) 144.12
P (s) 141.64
O (s) 138.28
N (s) 137.37
M (s) 134.21
L (d) 132.80
K (s) 129.47
J (s) 128.16
I (s) 124.97
H (s) 123.45
G (s) 122.92
F (s) 121.78
E (s) 120.85
D (s) 119.52
C (s) 115.73
B (s) 113.42
A (s) 107.03
f1 (ppm)

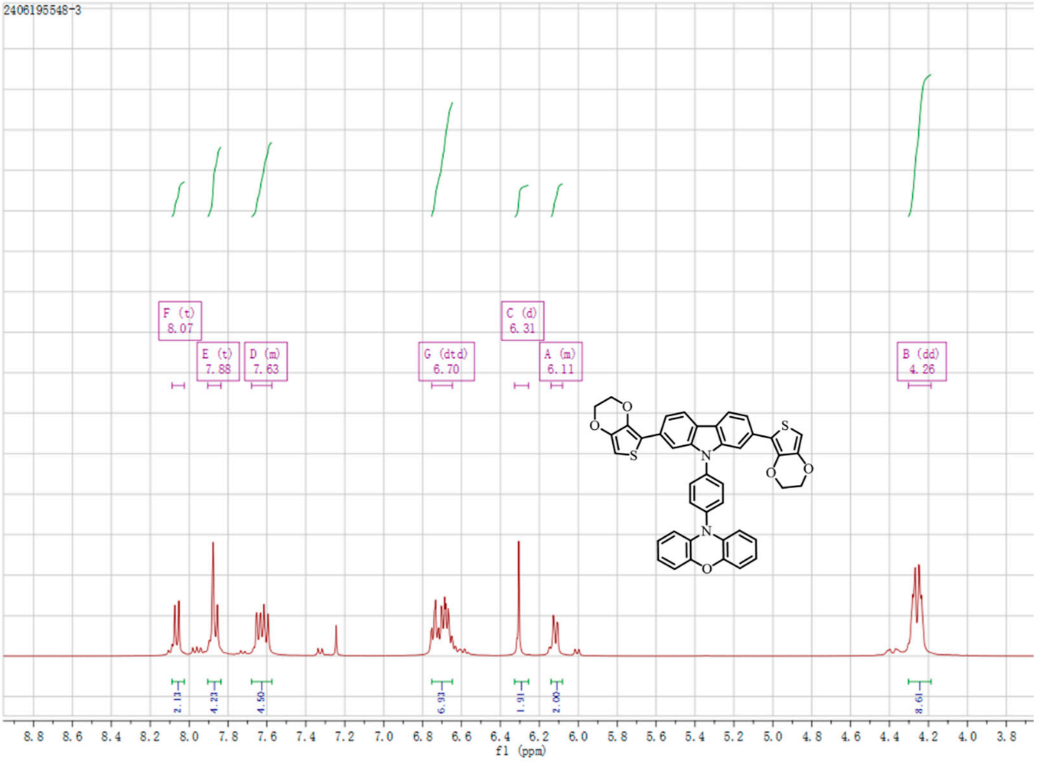

2406195548-3
F (t) 8.07
E (t) 7.88
D (m) 7.63
G (dtd) 6.70
C (d) 6.31
A (m) 6.11
B (dd) 4.26
2.13
4.23
4.50
6.93
1.91
2.00
8.61
f1 (ppm)

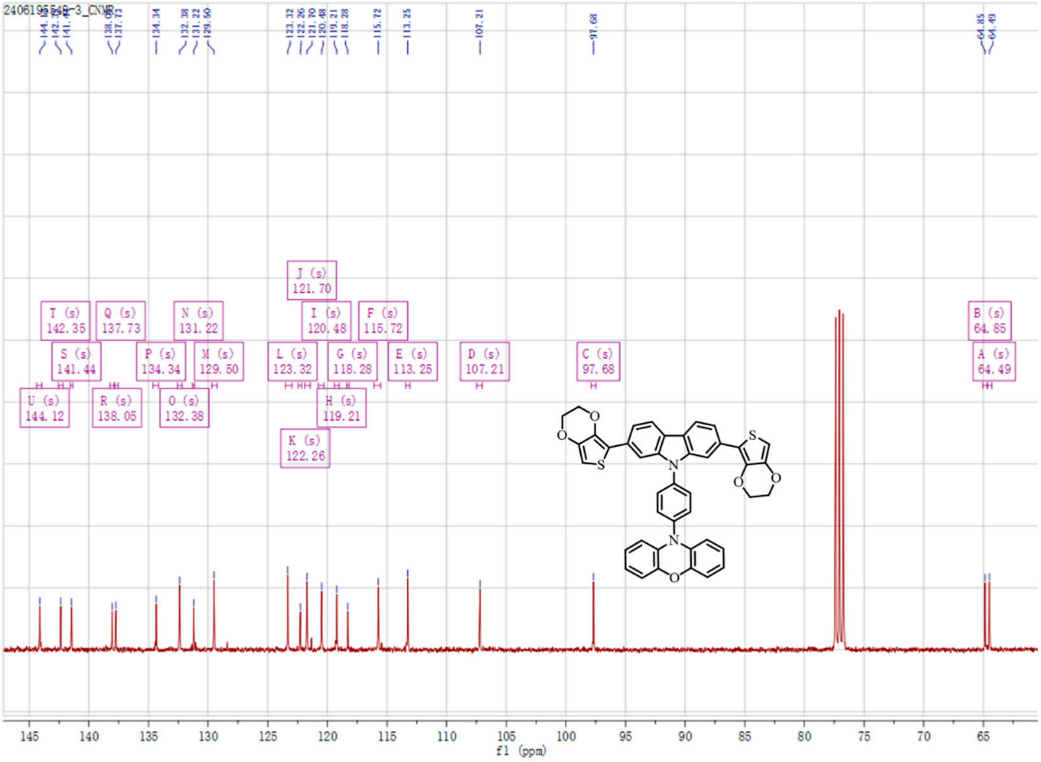

Figure. S1. The ^{1}H and ^{13}C NMR spectra of intermediate and monomers (DTCP and DDCP) dissolved in $CDCl_3$

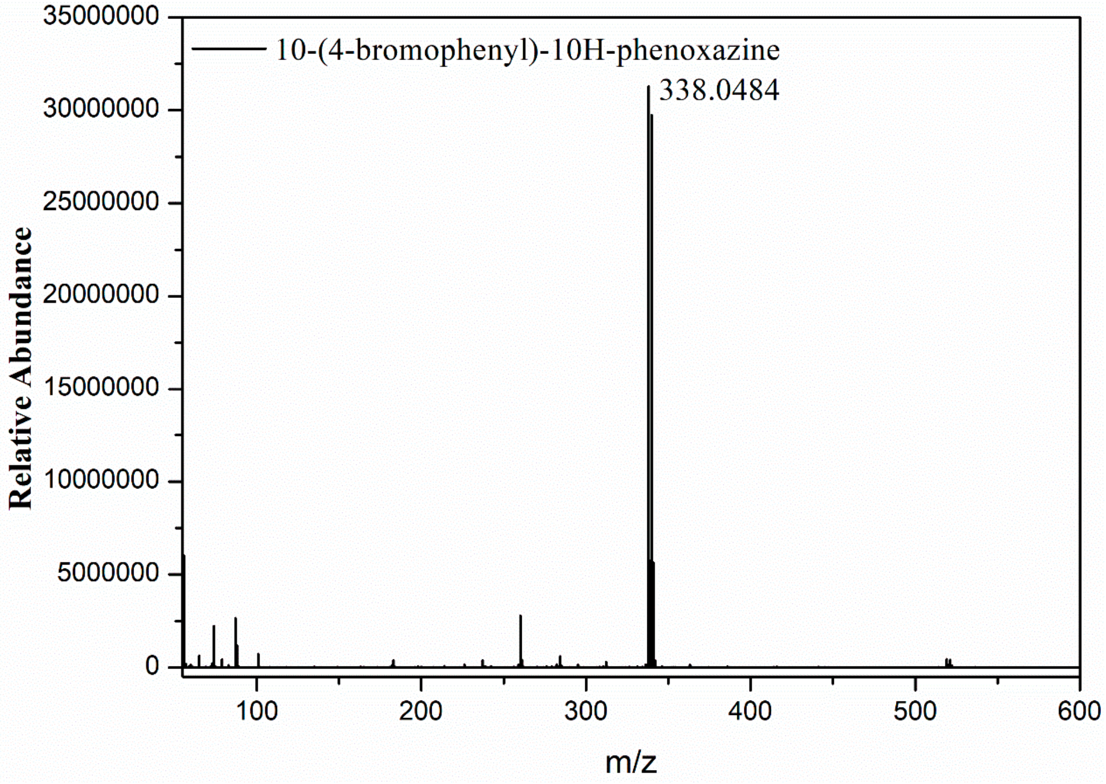

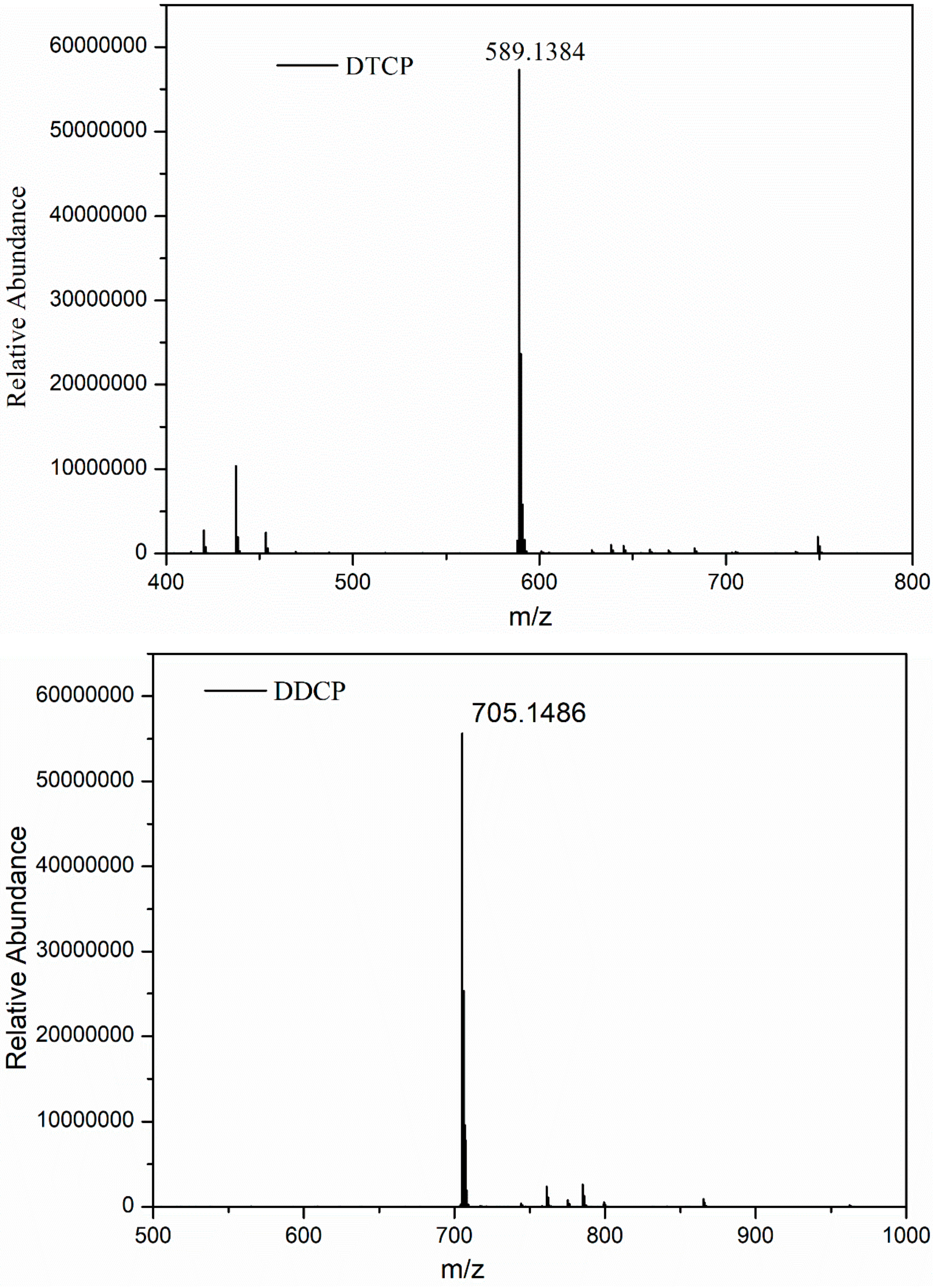

Figure S2. The high resolution mass spectrometry spectra of 10-(4-bromophenyl)-10H-phenoxazine, DTCP and DDCP

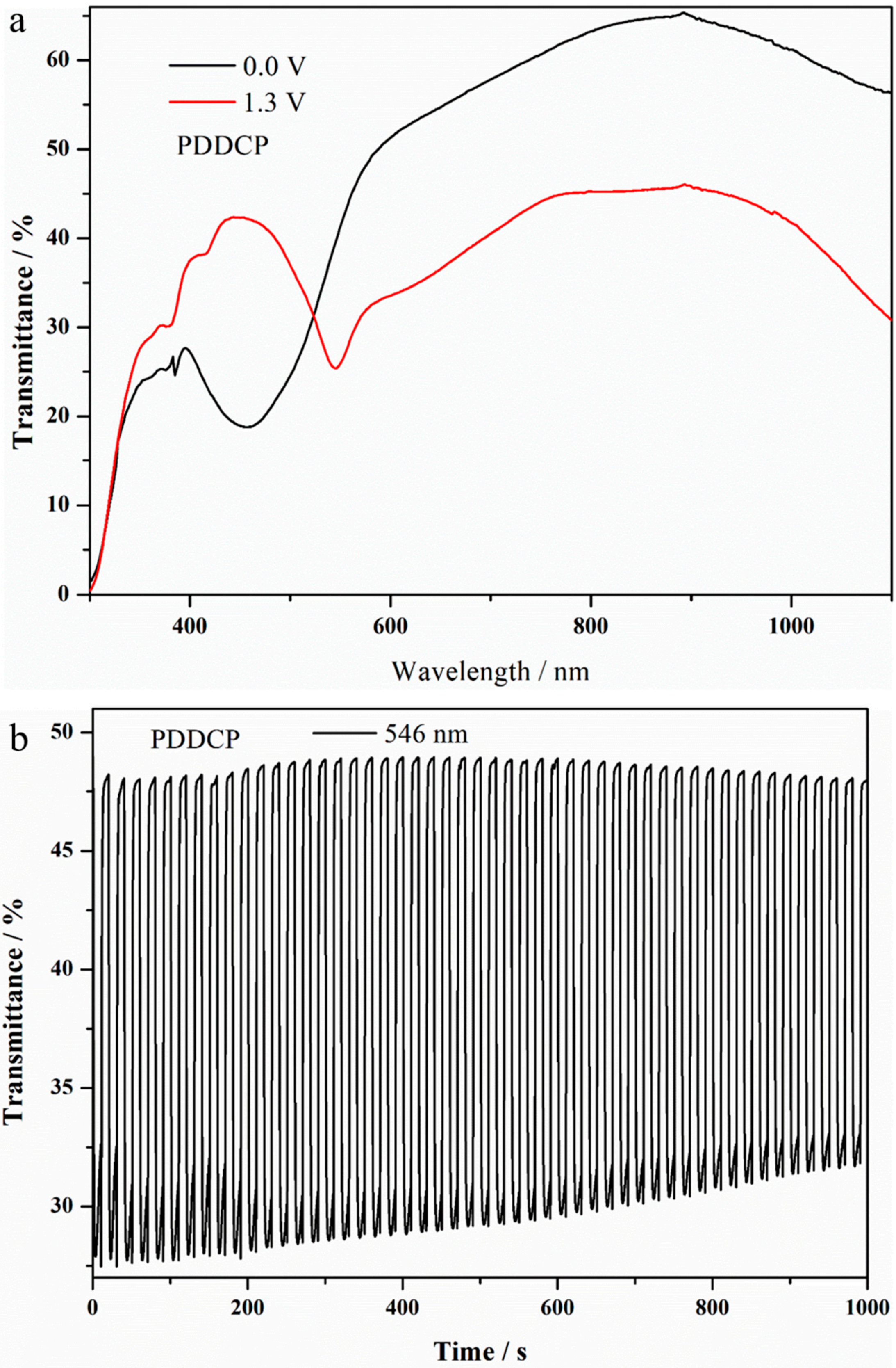
a
0.0 V
1.3 V
PDDCP
Transmittance / %
Wavelength / nm
0
10
20
30
40
50
60
400
600
800
1000
b
PDDCP
546 nm
Transmittance / %
Time / s
30
35
40
45
50
0
200
400
600
800
1000

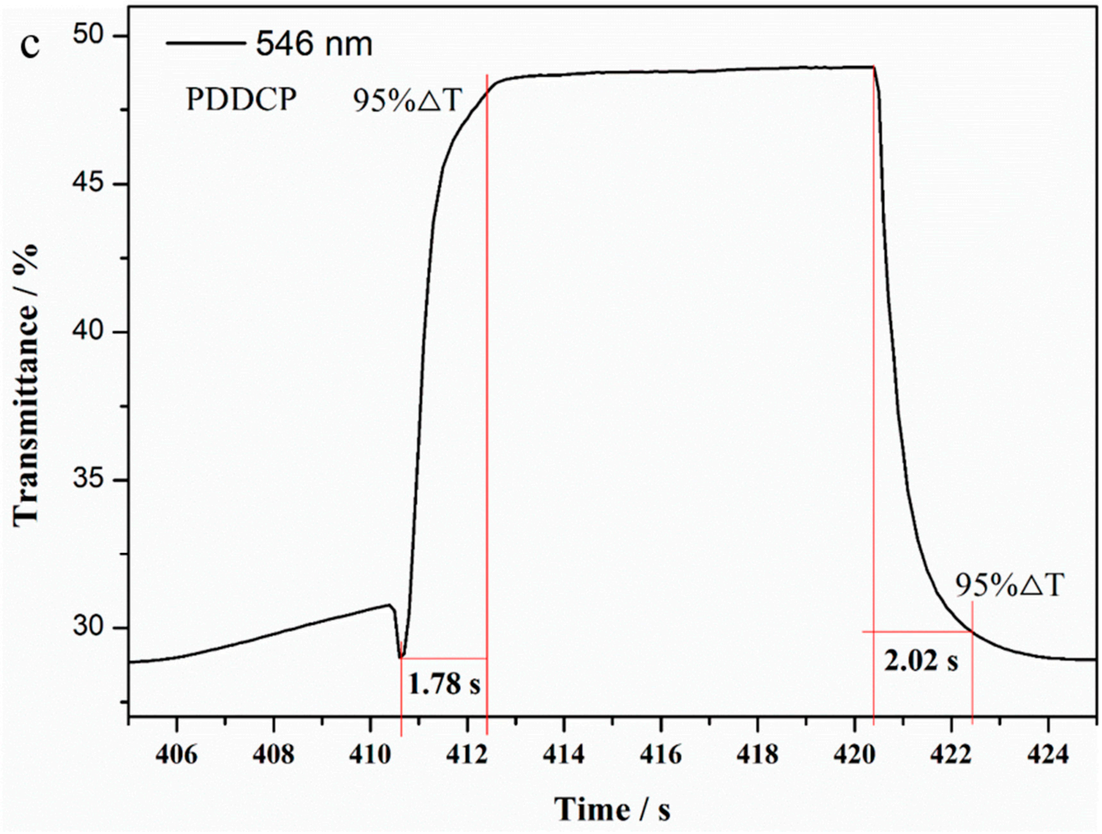

Figure S3. (a) The relationship between transmittance and different wavelengths of PDDCP films at 0.0 and 1.3 V; (b) Transmittance-time profiles of PDDCP films monitored at different absorption maxima under the applied potential between 0.0 V and 1.3V with the switching time of 10 s; (c) Calculated switching time of PDDCP at 546 nm.

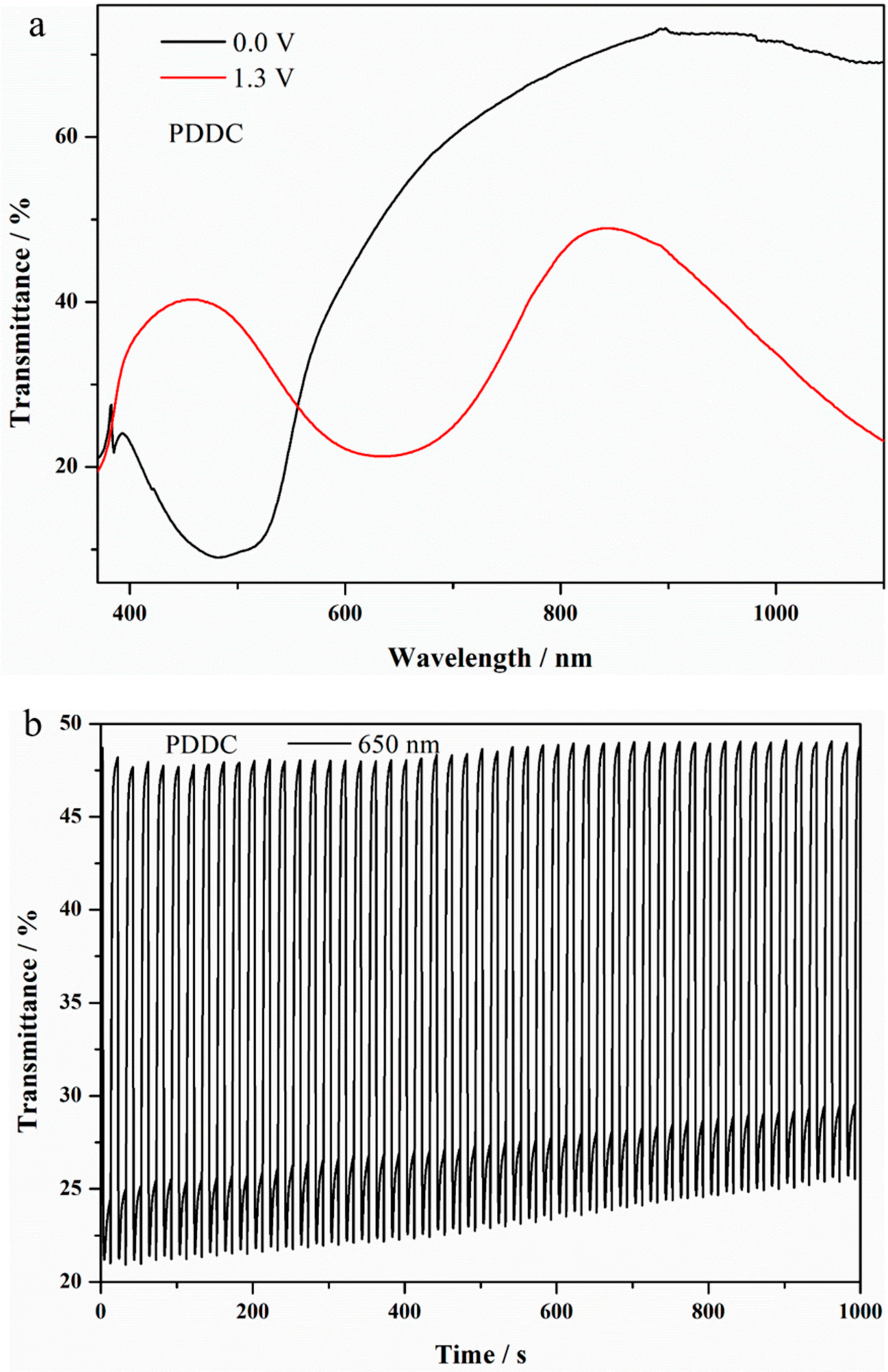
a
0.0 V
1.3 V
PDDC
Transmittance / %
20
40
60
400
600
800
1000
Wavelength / nm
b
PDDC
650 nm
Transmittance / %
20
25
30
35
40
45
50
0
200
400
600
800
1000
Time / s

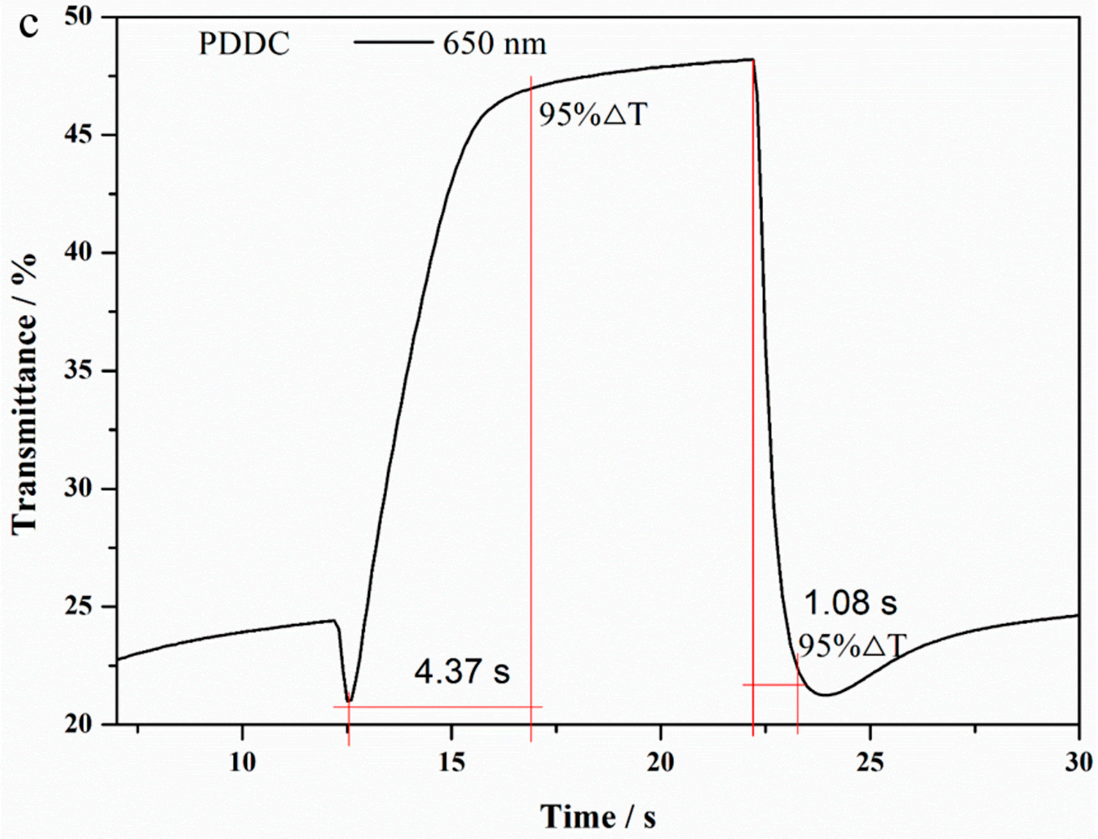

Figure S4. (a) The relationship between transmittance and different wavelengths of PDDC films at 0.0 and 1.3 V; (b) Transmittance-time profiles of PDDC films monitored at different absorption maxima under the applied potential between 0.0 V and 1.3 V with the switching time of 10 s; (c) Calculated switching time of PDDC at 650 nm.

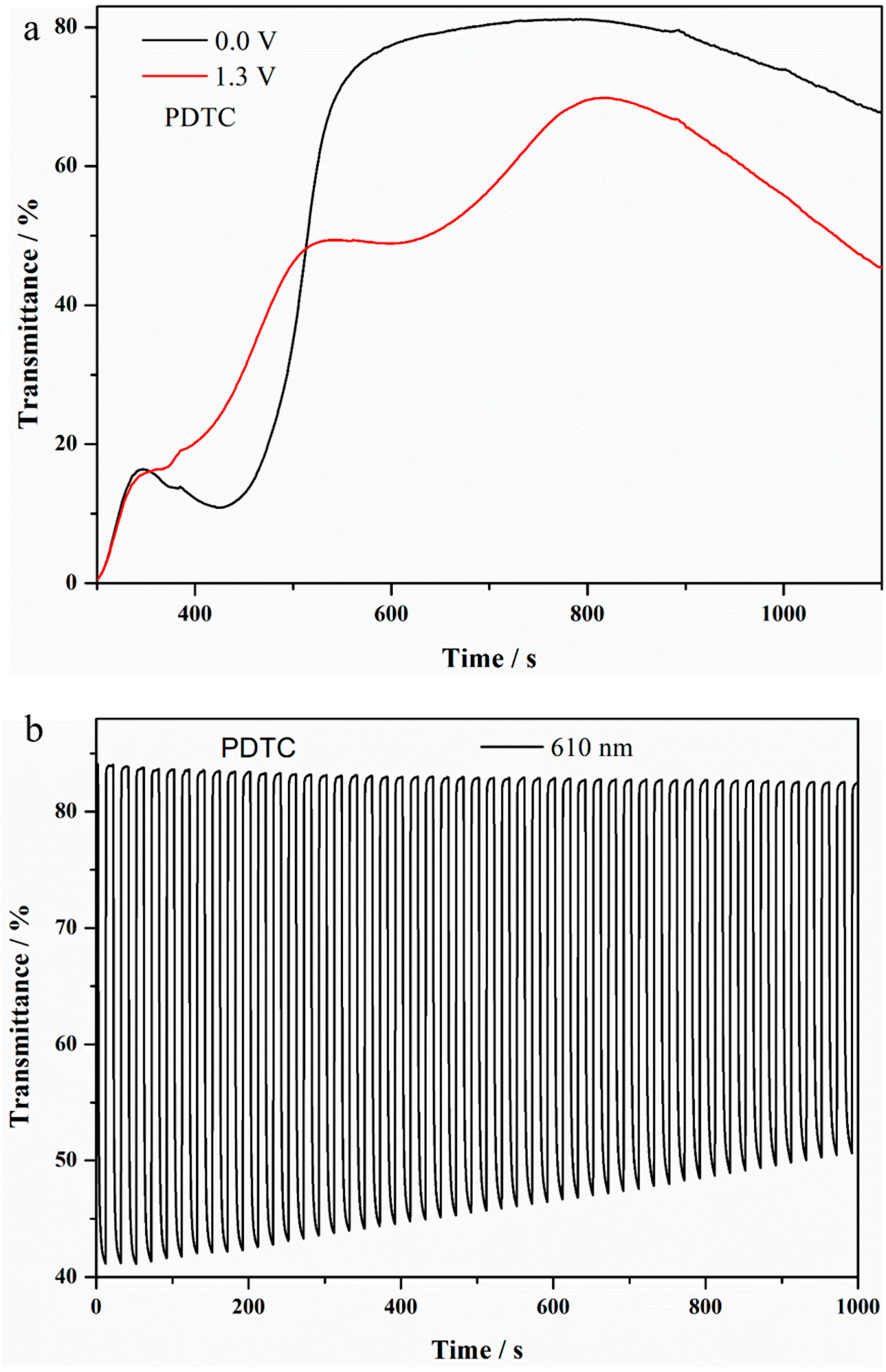
a
0.0 V
1.3 V
PDTC
Transmittance / %
Time / s
b
PDTC
610 nm
Transmittance / %
Time / s

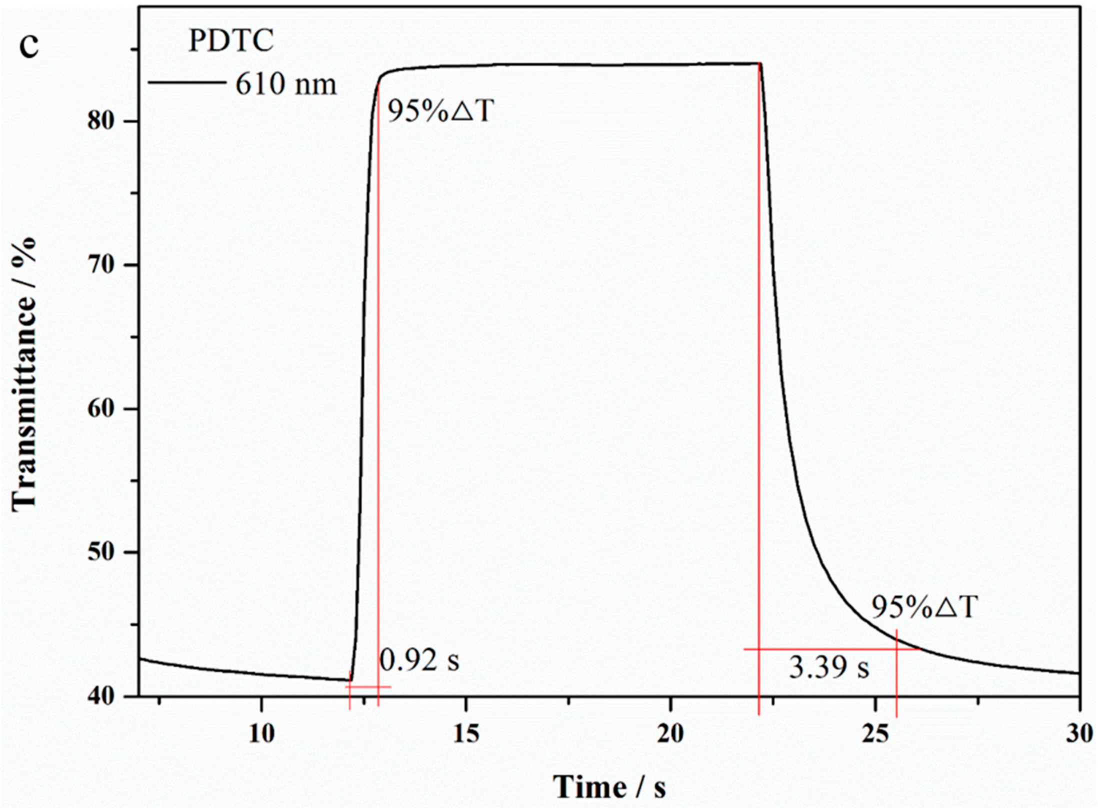

Figure S5. (a) The relationship between transmittance and different wavelengths of PDTC films at 0.0 and 1.3 V; (b) Transmittance-time profiles of PDTC films monitored at different absorption maxima under the applied potential between 0.0 V and 1.3 V with the switching time of 10 s; (c) Calculated switching time of PDTC at 610 nm.
